# Prospective blinded interim evaluation of a portable radiofrequency device TES HT to support triage in emergency department patients with mild head trauma or acute neurological symptoms

**DOI:** 10.1186/s12873-026-01676-8

**Published:** 2026-07-07

**Authors:** Adriana Modestina Conte, Fabrizio Alfieri, Angelo Localzo, Esaù Russo, Iole Cucinotto, Alessandro Trovato, Riccardo Gualtieri, Ettore Laccetti, Antonia Sorbo

**Affiliations:** 1 Pineta Grande Hospital, Castel Volturno, Italy; 2 B&B s.r.l, Casalnuovo di Napoli, Italy; 3 Independent Physician, Milan, Italy

**Keywords:** Emergency department, Mild head trauma, Acute neurological symptoms, Intracranial abnormalities, Head computed tomography, Diagnostic accuracy, Triage-support tool, Non-invasive radiofrequency device, Mild traumatic brain injury, TES HT100

## Abstract

**Background:**

Emergency Department (ED) evaluation of patients presenting with mild head trauma or acute neurological symptoms is common and often requires rapid exclusion of intracranial pathology. Although computed tomography (CT) is the diagnostic reference standard, its widespread use raises concerns regarding radiation exposure, cost, and ED workflow efficiency. TES HT100 is a portable, non-invasive radiofrequency-based device intended to assist early triage decisions. We report the prespecified interim analysis of a prospective validation study conducted in an ED population.

**Methods:**

Between December 2024 and January 2026, adult patients were enrolled at the Emergency Department of Pineta Grande Hospital, Castel Volturno, Italy. In this single-center, blinded diagnostic accuracy study, participants were included in two predefined cohorts: mild head trauma and non-traumatic acute neurological symptoms. TES HT100 provides a binary output and was compared with radiologist-interpreted head CT as the reference standard. Sensitivity, specificity, predictive values, likelihood ratios, accuracy, and balanced accuracy were calculated with 95% confidence intervals. Additional exploratory analyses stratified CT findings into acute clinically relevant abnormalities and chronic/incidental intracranial abnormalities without changing the prespecified primary endpoint. Robustness analyses assessed the impact of excluded or non-evaluable cases. Trial registration: ClinicalTrials.gov Identifier NCT07490418, registered on 23 March 2026.

**Results:**

The interim analysis included 301 patients (151 trauma; 150 neurological symptoms). CT-positive prevalence was 52.2%, consistent with the CT-imaged Emergency Department population and the protocol-defined broad intracranial abnormality endpoint. Overall sensitivity was 96.2% (95% CI 91.9–98.2), specificity 82.6% (75.6–88.0), PPV 85.8%, NPV 95.2%, and accuracy 89.7%. The positive likelihood ratio was 5.54 and the negative likelihood ratio 0.05. Exploratory category-specific analyses showed sensitivity and NPV of 96.6% and 98.4%, respectively, for acute clinically relevant abnormalities, and 96.0% and 96.0%, respectively, for chronic/incidental abnormalities. Specificity was lower in the acute-target analysis (50.6%), supporting interpretation of TES HT100 as a structural-abnormality triage signal rather than a lesion-specific acute-pathology classifier.

**Conclusions:**

In this interim evaluation, TES HT100 demonstrated high sensitivity and a low negative likelihood ratio compared with CT in ED patients with head trauma or acute neurological symptoms. These findings support further evaluation of TES HT100 as a triage-support adjunct in CT-imaged ED populations. Exploratory acute/chronic analyses provide additional clinical context but should not be interpreted as evidence of lesion-specific performance for acute abnormalities or as justification for replacing CT.

**Supplementary Information:**

The online version contains supplementary material available at https://doi.org/10.1186/s12873-026-01676-8.

## Background

Emergency department (ED) assessment of adults presenting with mild head trauma or acute neurological symptoms is a common and resource-intensive component of emergency care. Mild traumatic brain injury accounts for most traumatic brain injury presentations, and the burden of traumatic brain injury remains substantial across regions, with systematic reviews documenting high incidence globally and throughout Europe [1, 2].

In parallel, acute neurological presentations such as suspected stroke, transient focal deficits, acute confusion, speech disturbance, balance impairment, or syncope frequently require urgent evaluation to exclude intracranial pathology. Stroke alone remains a major global health problem and a leading cause of death and disability worldwide, reinforcing the need for timely identification of patients who may require neuroimaging [3].

Head computed tomography (CT) is widely used as the reference imaging modality for the rapid detection of acute intracranial hemorrhage and other structural brain abnormalities in the ED. However, the threshold for CT is often low in routine practice, particularly when symptoms are non-specific, risk factors are present, or clinical observation alone is felt to be insufficiently reassuring. Recent evidence suggests that CT overuse in mild head injury remains substantial across healthcare settings, with a pooled estimate of about 27% in one systematic review and marked variability across countries and regions [4].

Although CT is indispensable in many patients, its widespread use has important consequences. These include radiation exposure, additional costs, and pressure on already constrained ED workflows [5]. More broadly, ED crowding is an international systems-level challenge associated with treatment delays, reduced efficiency, and worse patient outcomes [6].

Clinical decision rules such as the Canadian CT Head Rule and the New Orleans Criteria have improved the standardisation of imaging decisions in selected trauma populations [7, 8]. However, uncertainty remains in routine ED practice, especially among older adults, patients receiving antithrombotic therapy, and patients with transient, mixed, or non-specific neurological symptoms. In these settings, there remains a practical need for adjunctive tools that can support early triage without replacing CT or overriding clinical judgement.

TES HT100 is a portable, non-invasive device that uses low-power radiofrequency electromagnetic waves to detect alterations in tissue dielectric properties associated with intracranial structural abnormalities. It provides a binary result within a few minutes and does not use ionising radiation. Preliminary early-phase reports on the same device have suggested encouraging diagnostic performance in both stroke-related intracranial lesions and mild traumatic brain injury populations, supporting further prospective clinical validation in real-world emergency settings [9, 10].

The TESCO study (CLV-01-TES HT) was designed as a prospective, blinded diagnostic accuracy study in the emergency department setting. It enrolled two clinically relevant cohorts: adults presenting with mild head trauma and adults presenting with suspected non-traumatic acute neurological symptoms who were not in a critical condition. This manuscript reports the prespecified first interim diagnostic accuracy analysis. The interim analysis was planned in the original protocol to provide an early protocol-defined assessment of diagnostic performance during the single-center phase of the study, before completion of the broader multicenter program; it was not intended for early stopping, safety-driven trial modification, or post hoc hypothesis generation.

## Methods

### Study design

The TESCO study (CLV-01-TES HT) is an ongoing prospective, single-center, blinded diagnostic accuracy study conducted in the Emergency Department (ED) of Pineta Grande Hospital, Castel Volturno, Italy. The study was designed to clinically evaluate the diagnostic performance of TES HT100 as a rapid, non-invasive screening and triage-support tool for the detection of intracranial abnormalities in ED patients, and not as a replacement for head CT, which remained the definitive diagnostic imaging standard throughout the study. The study includes two predefined cohorts: patients with mild head trauma and patients presenting with suspected non-traumatic acute neurological symptoms not requiring immediate life-saving intervention. Head CT, interpreted according to routine clinical practice, served as the reference standard. A prespecified interim analysis was planned once each cohort reached at least 150 enrolled participants. This manuscript reports the first interim diagnostic accuracy analysis conducted according to this predefined rule. The study design and reporting follow the STARD 2015 recommendations for diagnostic accuracy studies.

### Protocol status and amendments

The interim analysis reported in this manuscript was conducted according to Protocol version 1.1, which was the version in force at the time of the interim data cut-off. Prospective public registration on ClinicalTrials.gov was completed after study initiation because of procedural and administrative delays during preparation of the registry submission and subsequent requests from the registry reviewers to adapt protocol information to the ClinicalTrials.gov format. However, the study objectives, eligibility criteria, endpoints, and interim analysis plan were already defined in the ethics-approved original protocol before enrollment began and were not changed on the basis of interim results. A subsequent protocol amendment was approved in February 2026 after the interim data cut-off. This amendment converted the study into a multicenter study and clarified selected operational aspects of study conduct, but it did not modify the study objectives, eligibility criteria, endpoints, or analytical approach used for the interim analysis presented herein.

### Study setting

At the time of the interim analysis, recruitment had been conducted at the Emergency Department of Pineta Grande Hospital, Castel Volturno, Italy, a tertiary-care center. The interim analysis recruitment period covered December 2024 to January 2026. The study was approved by Comitato Etico Campania 3 (opinion CLV-01-TES HT; minutes no. 10/24 dated 24 June 2024; Protocol No. 00026087 dated 23 July 2024). Written informed consent was obtained from all participants prior to enrollment.

### Participants

Adult patients ( > = 18 years) presenting to the ED were eligible for inclusion if they met criteria for one of two predefined cohorts and provided written informed consent. Cohort A (mild head trauma) included adults presenting to the ED with mild head trauma, operationally defined for this report as head injury with a Glasgow Coma Scale (GCS) score of 13–15 at presentation and without clinical features requiring immediate advanced trauma or neurosurgical management [11]. Cohort B (non-traumatic neurological symptoms) included adults presenting with suspected non-traumatic acute neurological symptoms not in a critical or life-threatening condition, including syncope, spatial disorientation and/or balance disturbance, speech disturbance and/or facial hemiparesis, visual alterations, memory loss, and/or confusion.

Exclusion criteria for both cohorts were age under 18 years, pregnancy, metallic cranial implants, plates or prostheses, diagnosed skull fracture, critical clinical condition, inability to provide informed consent, or severe head trauma. For the purposes of this report, severe head trauma was operationally considered to include patients presenting with GCS ≤ 8 and/or requiring immediate life-saving or neurosurgical intervention.

Enrollment took place within the routine ED diagnostic pathway and was restricted to patients for whom non-contrast head CT was clinically indicated according to usual care. Accordingly, the study population should not be interpreted as an unselected ED screening population, but rather as a clinically enriched cohort undergoing neuroimaging assessment.

### Index test

The index test was TES HT100, a portable, non-invasive device that uses low-power, non-ionizing radiofrequency electromagnetic waves to detect intracranial abnormalities through alterations in dielectric tissue properties.

The device provides a binary output (positive/negative). Each examination lasts approximately 5 min, including about 4 min for signal acquisition and about 1 min for automated processing. TES examinations were performed by trained healthcare personnel, blinded to CT findings at the time of testing, according to the study procedures and without altering standard clinical care. The TES result was not used for clinical decision-making. Examinations affected by significant motion artifacts or technical issues preventing reliable interpretation were classified as non-evaluable.

### Reference standard

The reference standard was the radiologist-interpreted non-contrast head computed tomography (CT), performed according to routine clinical practice. For diagnostic accuracy analyses, participants were classified as CT-positive if at least one intracranial structural abnormality was reported on CT, irrespective of whether the finding was acute, chronic, traumatic, non-traumatic, symptomatic, incidental, or of uncertain immediate emergency relevance. This protocol-defined approach was intentionally adopted to reflect the current stage of development and intended use of TES HT100, which at present is designed to distinguish a structurally normal brain from a brain with structural intracranial abnormalities, rather than to classify specific lesion subtypes. The CT-positive category therefore included acute hemorrhagic lesions, ischemic lesions, calcifications, cystic or nodular lesions, chronic cerebrovascular changes, and sequelae of previous intracranial events when reported as intracranial abnormalities on routine CT. Extracranial findings without associated intracranial structural abnormality were classified as CT-negative for diagnostic performance calculations. Radiologists interpreting CT examinations were blinded to TES results.

For supplementary exploratory analyses, CT-positive findings were further categorized as acute clinically relevant abnormalities or chronic/incidental intracranial abnormalities according to the radiology report and subsequent clinical classification. This categorization was performed by consensus among the radiologist investigators who are co-authors of the study. When both acute and chronic/incidental findings were present in the same participant, the participant was included in both category-specific analyses.

### Study procedures and blinding

TES HT100 was performed as an adjunctive study procedure within the ED workflow and was not used to defer, replace, or delay standard assessment or CT imaging. TES examinations did not influence the clinical decision to perform CT imaging. According to protocol, the interval between TES and CT was not to exceed 24 h. In clinical practice during the interim study period, TES was generally performed after CT within the same ED episode and within a shorter operational interval. To minimize interpretation bias, TES was performed by trained personnel without access to the CT findings, while the radiologist interpreting the CT remained blinded to TES results. TES results were released to the clinical team only after CT reporting had been finalized and recorded.

### Outcomes

The primary outcome was the diagnostic performance of TES HT100 compared with head CT, assessed through sensitivity and specificity with 95% confidence intervals. Secondary outcomes included positive and negative predictive values, likelihood ratios and prespecified subgroup analyses.

### Interim analysis population

The study protocol prespecified an interim analysis once both study cohorts reached at least 150 enrolled participants. At the interim data cut-off (21 January 2026), a total of 321 participants had been enrolled. Three TES examinations were non-evaluable because of motion-related artifacts, and one participant did not undergo CT imaging. These cases were excluded from paired diagnostic accuracy analyses. The interim analysis set therefore included 301 participants, comprising 151 participants in Cohort A and the first 150 consecutive eligible and evaluable participants in Cohort B.

### Statistical analysis

Diagnostic performance was assessed by cross-tabulation of TES HT100 results against the CT reference standard. Sensitivity, specificity, positive predictive value (PPV), negative predictive value (NPV), overall accuracy, and balanced accuracy were calculated with 95% confidence intervals. Confidence intervals for proportions were calculated using the Wilson method. Positive and negative likelihood ratios (LR + and LR-) were computed with confidence intervals derived on the logarithmic scale [12]. Balanced accuracy was calculated as the arithmetic mean of sensitivity and specificity. Cohen’s kappa was calculated as a supplementary measure of agreement between TES HT100 and CT classification.

Additional exploratory analyses were performed by stratifying CT findings into acute clinically relevant abnormalities and chronic/incidental intracranial abnormalities. These analyses were conducted to further characterize device performance across different categories of intracranial findings and were considered supplementary to the prespecified primary endpoint based on overall intracranial structural abnormalities. Because acute and chronic/incidental findings could co-occur in the same CT examination, category-specific analyses were interpreted as target-condition analyses rather than mutually exclusive subgroup analyses. Number needed to diagnose (NND) was calculated as 1/(sensitivity + specificity − 1), corresponding to the inverse of Youden’s index.

Prespecified subgroup analyses were performed using two-sided Fisher’s exact tests. A robustness (tipping-point) sensitivity analysis evaluated the potential impact of excluded cases under conservative assumptions. All statistical tests were two-sided, and p-values < 0.05 were considered statistically significant for exploratory subgroup comparisons.

### Role of the sponsor

The study was developed by B&B Srl in collaboration with the principal investigator and the radiology team of Pineta Grande Hospital. The study database is maintained by Pineta Grande Hospital. Statistical analyses for this interim report were performed by B&B Srl under the methodological and clinical supervision of Iole Cucinotto and the principal investigator, based on a study database that was reviewed and checked by them before the final analyses were conducted. The reported analyses, data summaries, and their clinical interpretation were subsequently shared with and reviewed by the co-authors for consistency with the study protocol, endpoint definitions, and study findings. No separate formal third-party statistical validation was performed for this interim report. CT examinations were interpreted by radiologists blinded to TES results, and TES results were not used for clinical decision-making.

### Ethics

The study was approved by Comitato Etico Campania 3 (opinion CLV-01-TES HT; minutes no. 10/24 dated 24 June 2024; Protocol No. 00026087 dated 23 July 2024). Written informed consent was obtained from all participants before any study-specific procedure. The study was registered on ClinicalTrials.gov (Identifier: NCT07490418; registered on 23 March 2026).

### Reporting standard

This manuscript was prepared with reference to the STARD 2015 recommendations for reporting diagnostic accuracy studies.

## Results

### Study population and interim analysis cohort

**Fig. 1:**
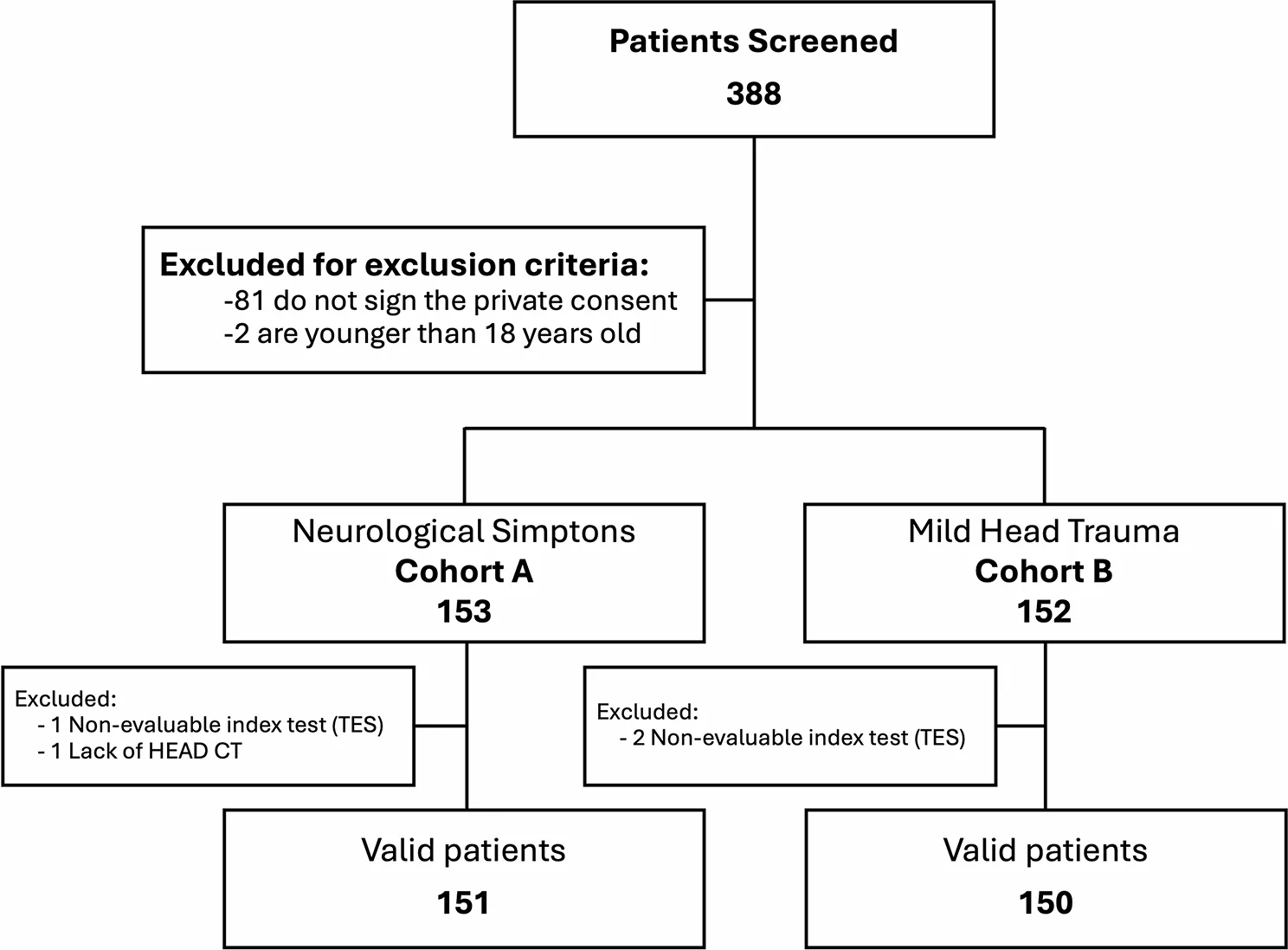
STARD-style flow diagram

During the interim study period, 301 adult patients with paired TES and head CT examinations were included in the prespecified interim analysis (151 mild head trauma; 150 neurological symptoms). The overall prevalence of CT-positive findings was 52.2% (157/301). The study population had a mean age of approximately 63 years (median 67), 55.1% of participants were aged > = 65 years, and 55.8% were male. The flow of participants from enrollment to inclusion in the interim diagnostic accuracy set is shown in Fig. 1. Baseline demographic and clinical characteristics are summarized in Table 1.

**Table 1:** Baseline characteristics (interim analysis set, *N* = 301)

Variable	Overall (*N* = 301)	Cohort A (*n* = 151)	Cohort B (*n* = 150)
Female, n (%)	133 (44.2%)	73 (48.3%)	60 (40.0%)
Male, n (%)	168 (55.8%)	78 (51.7%)	90 (60.0%)
Mean age (years)	~ 63	~ 60	~ 67
Median age (years)	67	63	70
Age ≥ 65 years, n (%)	166 (55.1%)	72 (47.7%)	94 (62.7%)

TES HT100 demonstrated sensitivity of 96.2% (95% CI 91.9–98.2), specificity of 82.6% (95% CI 75.6–88.0), PPV of 85.8%, NPV of 95.2%, overall accuracy of 89.7%, LR + of 5.54, and LR- of 0.05. The very low LR- is consistent with a strong rule-out profile in the protocol-defined study population [12]. The confusion matrix and the overall diagnostic performance estimates are presented in Tables 2A and 2B.

**Table 2A:** Confusion matrix (overall population, *N* = 301)

	CT Positive	CT Negative	Total
TES Positive	151	25	176
TES Negative	6	119	125
Total	157	144	301

**Table 2B:** Diagnostic performance (overall population)

Metric	Estimate	95% CI
Sensitivity	96.2%	91.9–98.2
Specificity	82.6%	75.6–88.0
Positive Predictive Value	85.8%	79.9–90.2
Negative Predictive Value	95.2%	89.9–97.8
Accuracy	89.7%	85.7–92.6
LR+	5.54	3.87–7.92
LR−	0.05	0.02–0.10

### Cohort-specific performance

In the neurological symptoms cohort (*n* = 150), sensitivity was 96.55% and specificity was 87.30%. In the mild head trauma cohort (*n* = 151), sensitivity was 95.71% and specificity was 79.01%. Sensitivity remained consistently high across age and sex strata. Exploratory subgroup diagnostic performance compared with cohort-level and overall performance is summarized in Table 3.

**Table 3:** Exploratory subgroup diagnostic performance compared to cohort performance and overall performance

Cohort	*N*	Prev (CT+)	Sens (95% CI)	Spec (95% CI)	PPV (95% CI)	NPV (95% CI)	Acc (95% CI)	LR+	LR-
Overall population	301	52.16%	96.18% (91.9–98.2)	82.6% (75.6–88.0)	85.8% (79.9–90.2)	95.2% (89.9–97.8)	89.7% (85.7–92.6)	5.54	0.05
Mild head trauma (A)	151	46.4%	95.7% (88.1–98.5)	79.0% (68.9–86.5)	79.8% (70.0–87.0)	95.5% (87.6–98.5)	86.7% (80.4–91.3)	4.56	0.05
Neurological symptoms (B)	150	58.0%	96.5% (90.3–98.8)	87.3% (76.9–93.4)	91.3% (83.8–95.5)	94.8% (85.9–98.2)	92.7% (87.3–95.9)	7.60	0.04
Elderly ( > = 65)	166	63.9%	95.3% (89.4–98.0)	91.7% (81.9–96.4)	95.3% (89.4–98.0)	91.7% (81.9–96.4)	94.0% (89.3–96.7)	11.43	0.05
Adult (18–64)	135	37.8%	98.04% (89.7–99.6)	76.2% (66.1–84.0)	71.43% (59.9–80.7)	98.5% (91.8–99.7)	84.4% (77.4–89.6)	4.12	0.03
Male	168	51.8%	96.55% (90.3–98.8)	82.7% (73.0-89.4)	85.7% (77.4–91.3)	95.7% (88.1–98.5)	89.9% (84.4–93.6)	5.59	0.04
Female	133	52.6%	95.7% (88.1–98.5)	82.54% (71.4–90.0)	85.9% (76.5–91.9)	94.5% (85.1–98.1)	89.5% (83.1–93.6)	5.48	0.05

### Exploratory analysis according to CT finding category

An exploratory stratified analysis was performed according to the nature of CT findings. Acute clinically relevant abnormalities were present in 58/301 participants (19.3%), chronic/incidental abnormalities in 126/301 participants (41.9%), and both categories in 27 participants (9.0%). As these categories were not mutually exclusive, the analyses should be interpreted as category-specific target-condition analyses rather than as mutually exclusive patient subgroups.

When acute clinically relevant abnormalities were considered as the target condition, TES HT100 showed TP = 56, FP = 120, FN = 2, and TN = 123, corresponding to sensitivity of 96.6% (95% CI 88.3–99.0), specificity of 50.6% (44.4–56.8), PPV of 31.8%, NPV of 98.4%, LR + of 1.96, LR- of 0.07, overall accuracy of 59.5%, balanced accuracy of 73.6%, and NND of 2.12.

When chronic/incidental intracranial abnormalities were considered as the target condition, TES HT100 showed TP = 121, FP = 55, FN = 5, and TN = 120, corresponding to sensitivity of 96.0% (95% CI 91.0-98.3), specificity of 68.6% (61.4–75.0), PPV of 68.8%, NPV of 96.0%, LR + of 3.06, LR- of 0.06, overall accuracy of 80.1%, balanced accuracy of 82.3%, and NND of 1.55.

These supplementary analyses provide additional clinical context regarding device performance across different categories of intracranial abnormalities while leaving unchanged the primary protocol-defined endpoint. Detailed category-specific diagnostic performance estimates are reported in Supplementary Table 1.

### Subgroup analyses

Exploratory subgroup analyses stratified by age and sex did not demonstrate statistically significant differences in sensitivity or specificity across predefined subgroups. In the elderly subgroup, the confidence intervals for sensitivity and PPV, as well as for specificity and NPV, are numerically identical because the 2 × 2 table is symmetrical with the same number of false-positive and false-negative results in that subgroup. The rule-out profile remained stable across both cohorts.

### Robustness analysis

A tipping-point sensitivity analysis was conducted to evaluate the potential impact of excluded or non-evaluable cases under conservative assumptions. Under extreme scenarios assuming misclassification of excluded cases, overall sensitivity and negative likelihood ratios remained within clinically acceptable ranges, supporting the robustness of the interim findings.

## Discussion

### Principal findings

In this prespecified interim analysis conducted in an ED setting, TES HT100 demonstrated high sensitivity and a very low negative likelihood ratio for the detection of protocol-defined intracranial abnormalities compared with CT, a metric that is particularly useful for translating diagnostic performance into post-test probability in clinical decision-making [12]. These findings support further evaluation of TES HT100 as a triage-support adjunct in CT-imaged ED populations, rather than as a replacement for CT. However, the results derive from a single-center interim cohort, do not address clinical impact or CT-use reduction, and should not be interpreted as evidence of lesion-specific performance for acute abnormalities requiring urgent treatment. Completion of the full study and further multicenter validation are warranted.

### Interpretation in the ED context

In real-world Emergency Departments, clinicians frequently evaluate patients with mild head injury or non-specific neurological symptoms who ultimately demonstrate negative imaging findings. Although CT remains indispensable for definitive diagnosis, the threshold for ordering imaging is often influenced by clinical uncertainty, patient age, anticoagulant use, and institutional practice patterns. In parallel, CT overuse in mild head injury remains substantial across healthcare settings, while ED crowding has been consistently associated with delays, inefficiency, and harm at the system level [4, 13]. 

In this context, where rapid risk stratification is essential, adjunctive tools capable of supporting early triage without replacing CT are of interest. This new technology may contribute to support early clinical decision-making to more efficient patient management without altering established imaging standard. The diagnostic profile observed in this interim cohort suggests that the device may contribute to identifying patients at low probability of intracranial abnormalities when integrated into existing clinical pathways. Importantly, this study was not designed to assess reduction in CT utilization, and no conclusions regarding imaging avoidance can be drawn from these data.

### Prevalence and case-mix considerations

The observed CT-positive prevalence of 52.2% is higher than typically reported in unselected mild head injury cohorts and should be interpreted in light of the study design. Enrollment was restricted to patients who underwent clinically indicated head CT in routine ED care, resulting in a clinically enriched population. In addition, the cohort was relatively old, with a mean age of approximately 63 years and more than half of participants aged > = 65 years, a context in which chronic cerebrovascular and other structural intracranial findings are more common. Finally, the protocol-defined CT-positive category intentionally included any intracranial structural abnormality reported on routine CT, whether acute, chronic, previous, or incidental.

This developmental endpoint was chosen because TES HT100 is currently intended to distinguish structurally normal from structurally abnormal brains, rather than to classify lesion subtype. Accordingly, the specificity reported in the primary interim analysis should not be interpreted as lesion-specific specificity for acute abnormalities requiring urgent management. The exploratory acute-versus-chronic/incidental analyses reported in this revision provide additional context but do not redefine the primary endpoint or establish definitive lesion-subtype performance.

### Exploratory interpretation of acute and chronic/incidental findings

Additional exploratory analyses according to CT finding category provided further insight into the clinical interpretation of device performance. When restricted to acute clinically relevant abnormalities, TES HT100 maintained very high sensitivity and negative predictive value, supporting its potential value as a rule-out triage-support tool in patients undergoing emergency neuroimaging. However, specificity was substantially lower in this setting, indicating that the device should not be interpreted as a lesion-specific diagnostic classifier for acute pathology.

The lower specificity and positive predictive value observed in the acute-target analysis are clinically important. They likely reflect the fact that TES-positive results may also be associated with chronic or incidental structural abnormalities that are outside the acute target condition and are therefore counted as false positives in that specific analysis. The corresponding analysis for chronic/incidental abnormalities showed similarly high sensitivity, supporting the hypothesis that TES HT100 primarily identifies the presence of structural intracranial abnormalities rather than distinguishing specific pathological subtypes.

### Robustness of findings

Subgroup analyses did not demonstrate significant variation in diagnostic performance across age and sex strata, and the rule-out profile remained stable across the two predefined clinical cohorts. Additionally, conservative tipping-point analyses suggested that the overall sensitivity and LR− remained within clinically acceptable ranges even under extreme assumptions regarding the small number of excluded or non-evaluable cases. These findings support the internal robustness of the interim results, while recognizing that definitive subgroup conclusions will require the full study population.

### Relation to previous evidence

The present interim results are also coherent with the early clinical literature available for the same technology. Preliminary reports on TES HT100 have suggested encouraging diagnostic performance both in patients with intracranial lesions related to stroke and in mild traumatic brain injury populations. The current study extends that early evidence by using a prospective blinded design, a larger interim sample, and a broader emergency department case mix that more closely reflects real-world triage conditions [9, 10]. 

### Limitations

Several limitations should be acknowledged. First, this is a single-center interim analysis and may not fully represent broader ED populations. Second, the sample size, although prespecified, remains limited relative to definitive validation studies. Third, the CT-positive definition intentionally included acute, chronic, and incidental intracranial abnormalities. Although exploratory category-specific analyses have been reported, these analyses were not part of the original protocol-defined primary endpoint structure and should therefore be interpreted as supplementary and hypothesis-generating. In particular, the acute-target analysis is affected by the fact that TES HT100 may detect chronic/incidental structural abnormalities that are counted as false positives when only acute clinically relevant abnormalities are considered as the target condition.

An additional limitation is that prospective public registration on ClinicalTrials.gov was completed after study initiation because of procedural and administrative delays and subsequent requests to adapt protocol information to the registry format. Although the study objectives, eligibility criteria, endpoints, and interim analysis plan had already been defined in the ethics-approved original protocol before enrollment began and were not changed on the basis of interim results, the timing of public registration should be acknowledged as a reporting limitation.

Furthermore, this investigation evaluated diagnostic accuracy rather than clinical impact. The study did not assess changes in CT ordering behavior, workflow efficiency, or patient outcomes. Larger multicenter studies will be necessary to determine the real-world impact of integrating such technology into ED decision pathways and to confirm the exploratory acute-versus-chronic/incidental findings in the completed study population.

### Future directions

Completion of the full planned study cohort and expansion to multicenter validation will be essential to confirm generalizability and to explore the potential role of this technology within structured triage algorithms. Future investigations should confirm performance according to lesion acuity and clinical relevance, and should assess the effect of integration into care pathways on workflow and patient management.

## Conclusions

In this prespecified interim analysis conducted in an ED setting, TES HT100 demonstrated high sensitivity and a very low negative likelihood ratio for the detection of protocol-defined intracranial abnormalities compared with CT in patients presenting with mild head trauma or acute neurological symptoms. Exploratory category-specific analyses showed maintained sensitivity and negative predictive value for both acute clinically relevant abnormalities and chronic/incidental abnormalities, but lower specificity when only acute findings were considered as the target condition. These findings support further evaluation of TES HT100 as a triage-support adjunct in CT-imaged ED populations, rather than as a replacement for CT or as a lesion-specific acute-pathology classifier. Completion of the full study and further multicenter validation are warranted.

## Supplementary Information

Below is the link to the electronic supplementary material.

Supplementary Material 1 : Supplementary Table 1. Exploratory diagnostic performance according to CT finding category

## Data Availability

The datasets generated and/or analyzed during the current study are not publicly available due to institutional and sponsor restrictions but are available from the corresponding author on reasonable request.
