# Supplementary material for: Prospective blinded interim evaluation of a portable radiofrequency device TES HT to support triage in emergency department patients with mild head trauma or acute neurological symptoms
Source: BMC Emerg Med. 2026 Jul 7;26:259. doi: 10.1186/s12873-026-01676-8 (PMC13637347; doi:10.1186/s12873-026-01676-8)
Supplement: Supplementary file 1 [file 12873_2026_1676_MOESM1_ESM.docx]

**Supplementary Table 1. Exploratory diagnostic performance according to CT finding category**

Category-specific analyses were supplementary to the prespecified primary endpoint based on overall intracranial structural abnormalities. Acute and chronic/incidental categories were not mutually exclusive; participants with both acute and chronic/incidental findings were included in both exploratory analyses.

| **Target condition** | **CT+ n/N (%)** | **TP** | **FP** | **FN** | **TN** | **Sensitivity (95% CI)** | **Specificity (95% CI)** | **PPV (95% CI)** | **NPV (95% CI)** | **LR+ (95% CI)** | **LR- (95% CI)** | **Accuracy** | **Balanced accuracy** | **NND** | **Cohen's kappa** |
| --- | --- | --- | --- | --- | --- | --- | --- | --- | --- | --- | --- | --- | --- | --- | --- |
| Overall CT-positive endpoint | 157/301 (52.2%) | 151 | 25 | 6 | 119 | 96.2% (91.9-98.2) | 82.6% (75.6-88.0) | 85.8% (79.9-90.2) | 95.2% (89.9-97.8) | 5.54 (3.87-7.92) | 0.05 (0.02-0.10) | 89.7% | 89.4% | 1.27 | 0.793 |
| Acute clinically relevant abnormalities | 58/301 (19.3%) | 56 | 120 | 2 | 123 | 96.6% (88.3-99.0) | 50.6% (44.4-56.8) | 31.8% (25.4-39.0) | 98.4% (94.4-99.6) | 1.96 (1.71-2.24) | 0.07 (0.02-0.27) | 59.5% | 73.6% | 2.12 | 0.266 |
| Chronic/incidental abnormalities | 126/301 (41.9%) | 121 | 55 | 5 | 120 | 96.0% (91.0-98.3) | 68.6% (61.4-75.0) | 68.8% (61.6-75.1) | 96.0% (91.0-98.3) | 3.06 (2.45-3.81) | 0.06 (0.02-0.14) | 80.1% | 82.3% | 1.55 | 0.612 |

**Abbreviations:** CT+, computed tomography-positive; TP, true positive; FP, false positive; FN, false negative; TN, true negative; PPV, positive predictive value; NPV, negative predictive value; LR+, positive likelihood ratio; LR-, negative likelihood ratio; NND, number needed to diagnose. NND was calculated as 1/(sensitivity + specificity - 1).
